# *In silico* comparative analysis of LRRK2 interactomes from brain, kidney and lung

**DOI:** 10.1016/j.brainres.2021.147503

**Published:** 2021-08-15

**Authors:** Amrita Verma, Kirsten Ebanks, Chi-Yee Fok, Patrick A. Lewis, Conceicao Bettencourt, Rina Bandopadhyay

**Affiliations:** a Reta Lila Weston Institute of Neurological Studies, Department of Clinical and Movement Neuroscience, UCL Queen Square Institute of Neurology, London WC1N 1PJ, United Kingdom; b Royal Veterinary College, Royal College Street, London NW10TV, United Kingdom; c Department of Neurodegenerative Disease and Queen Square Brain Bank, UCL Queen Square Institute of Neurology, London WC1N 1PJ, United Kingdom; d Aligning Science Across Parkinson’s (ASAP) Collaborative Research Network, Chevy Chase, MD, United States

**Keywords:** BINGO, *In silico*, Gene ontology, Gene expression, HIPPIE, LRRK2-interactome

## Abstract

Mutations in *LRRK2* are the most frequent cause of familial Parkinson’s disease (PD), with common *LRRK2* non-coding variants also acting as risk factors for idiopathic PD. Currently, therapeutic agents targeting LRRK2 are undergoing advanced clinical trials in humans, however, it is important to understand the wider implications of LRRK2 targeted treatments given that LRRK2 is expressed in diverse tissues including the brain, kidney and lungs. This presents challenges to treatment in terms of effects on peripheral organ functioning, thus, protein interactors of LRRK2 could be targeted *in lieu* to optimize therapeutic effects. Herein an *in-silico* analysis of LRRK2 direct interactors in brain tissue from various brain regions was conducted along with a comparative analysis of the LRRK2 interactome in the brain, kidney, and lung tissues. This was carried out based on curated protein–protein interaction (PPI) data from protein interaction databases such as HIPPIE, human gene/protein expression databases and Gene ontology (GO) enrichment analysis using Bingo. Seven targets (MAP2K6, MATK, MAPT, PAK6, SH3GL2, CDC42EP3 and CHGB) were found to be viable objectives for LRRK2 based investigations for PD that would have minimal impact on optimal functioning within peripheral organs. Specifically, MAPT, CHGB, PAK6, and SH3GL2 interacted with LRRK2 in the brain and kidney but not in lung tissue whilst LRRK2-MAP2K6 interacted only in the cerebellum and MATK-LRRK2 interaction was absent in kidney tissues. *CDC42EP3* expression levels were low in brain tissues compared to kidney/lung. The results of this computational analysis suggest new avenues for experimental investigations towards LRRK2-targeted therapeutics.

## Highlights

• *In silico* comparative analysis of LRRK2 direct interactors in brain, lung and kidney using Protein interaction databases and gene enrichment analysis.
• 7 promising therapeutic interactors identified: MAP2K6, MATK, MAPT, PAK6, SH3GL2, CDC42EP3, CHGB.
• Expression levels of *CHGB*, *MAPT*, and *SH3GL2* were low in kidney and lung compared to brain tissues.
• MAPT, CHGB, PAK6, SH3GL2 interacted with LRRK2 in the brain and kidney but not in the lungs.
• PAK6-LRRK2 interacted in all brain regions and MATK-LRRK2 interaction was absent in kidney.

## 1 Introduction

Parkinson’s disease (PD) is an age-related neurodegenerative disease affecting more than 1% of the population above 60 years with currently no curative therapies available (Tysnes and Storstein, 2017). PD is clinically characterized by abnormal postural reflexes, muscular rigidity, and bradykinesia, and is also accompanied by a range of non-motor symptoms such as cognitive impairment and depression (Han et al., 2018, Schapira et al., 2017). Neuropathologically PD is characterised by degeneration of dopaminergic neurons in *substantia nigra pars compacta*, and the formation of insoluble protein aggregates known as Lewy bodies, composed predominantly of alpha-synuclein (Spillantini et al., 1998).

Since 1997, mutations in several genes have been found to contribute to familial PD (Poewe et al., 2017). In 2004, point mutations in the Leucine-rich repeat kinase 2 (*LRRK2*) gene were discovered by two groups simultaneously (Paisán-Ruíz et al., 2004, Zimprich et al., 2004) and subsequently, *LRRK2* mutations have been linked to 5–8% of familial (Nichols et al., 2005) and 1–2% of sporadic PD cases (Gilks et al., 2005). Additionally, *LRRK2* variants are also a risk factor for idiopathic PD (Nalls et al., 2014). The G2019S mutation is the most common pathogenic mutation, however, a number of others have been described (e.g. R1441C, Y1699C, I2020T) (Gaig et al., 2006, Klein and Westenberger, 2012). Apart from PD, *LRRK2* has also been linked to cancer (Ruiz-Martínez et al., 2014), inflammatory bowel disorder (IBD) (Jostins et al., 2012), multibacillary leprosy (Zhang et al., 2009), and functionally with tuberculosis (Härtlova et al., 2018).

The large size and complexity of the LRRK2 protein is likely responsible for the relationship with a wide variety of human diseases. LRRK2 is a 286-kDa protein kinase that belongs to the ROCO protein family (Marín, 2006) and is composed of multiple domains. At its core, LRRK2 protein consists of ROC (Ras of complex proteins) GTPase domain, a COR (C-terminal of ROC) dimerization domain, and a kinase domain. It also consists of four tandem repeats domains N-terminal Armadillo, Ankyrin, Leucine-rich repeats, and a C-terminal WD40 fold (Marín, 2006). These domains form a large diversity of stable protein folds thus mediating a variety of protein–protein interactions (Gomez-Suaga et al., 2014). Interestingly, all segregating PD related mutations are found within the enzymatic core of LRRK2 (Tolosa et al., 2020). The G2019S mutation is associated with increased kinase activity and leads to cellular toxicity (Greggio et al., 2006). Moreover, a recent study using the proximity ligation assay technique has shown that LRRK2 kinase activity was enhanced in dopamine neurons from postmortem brains of idiopathic PD patients and also in two different sporadic PD mouse models (Di Maio et al., 2018). Therefore, the LRRK2 kinase domain has presented itself as a druggable target and indeed several kinase inhibitors are in a clinical trial for potential PD therapy (Zhao and Dzamko, 2019). Currently, phase II clinical trials are in progress through Denali Therapeutics (NCT04056689) for small molecule kinase inhibitors, and LRRK2 antisense technology trials through Ionis/Biogen (NCT03976349). However, the precise consequences of LRRK2 kinase inhibition in brain and peripheral tissues remains to be determined.

Previous work on LRRK2 targeted therapy in rodent models demonstrated debatable results. For example, LRRK2 deficiency led to the development of pathogenic phenotype in the lungs and kidney (Herzig et al., 2011, Hinkle et al., 2012, Tong et al., 2010). However, the administration of LRRK2 kinase inhibitors in rodents showed pathological changes in kidney but not in the lungs (Andersen et al., 2018, Herzig et al., 2011, Ness et al., 2013). A recent study however has shown that these kinase inhibitors have a reversible effect on the lung phenotype in non-human primates (Baptista et al., 2020). These exaggerated effects on peripheral organs might be because LRRK2 is ubiquitously expressed, with the highest expression levels observed in the kidney, lung, and monocytes.

LRRK2 protein function is linked with a wide variety of cellular events including vesicular trafficking, cytoskeletal function, autophagy, inflammation, and the regulation of the endo-lysosomal system (Plowey et al., 2008, Ramonet et al., 2011, Rudyk et al., 2019). A plethora of functional studies have been done on LRRK2 thus far, providing an extensive body of work to support *in silico* study. Previously computational analysis of LRRK2 protein–protein interaction (PPI) has been done based on the International Molecular Exchange (IMEx) consortium (Orchard et al., 2012) data from IntAct (Orchard et al., 2014) and BioGRID (Chatr-Aryamontri et al., 2017) to understand and find novel interactors of LRRK2 and its cellular pathway (Gloeckner and Porras, 2020, Manzoni et al., 2015, Porras et al., 2015). To date, however, comparative tissue specific *in silico* interactome analysis for LRRK2 has not been carried out. Here we propose a new method of potential drug target discovery which takes into account not only the possible therapeutic effects but also seeks to minimise unwanted effects in other tissues. Thus, we have analyzed the LRRK2 interactome in brain tissues majorly affected in PD; such as the substantia nigra, basal ganglia, frontal cortex, and anterior cingulate as well as the cerebellum as region unaffected by PD pathology (Braak et al., 2003, Dijkstra et al., 2014, Poewe et al., 2017). As previously detailed, drugs and treatments inhibiting LRRK2 to treat PD have been shown to induce pathologies in peripheral organs, particularly the kidney and lungs (Herzig et al., 2011, Hinkle et al., 2012, Tong et al., 2010). Therefore, we also investigated kidney and lung tissue, where LRRK2 is highly expressed, and conducted an *in silico* comparative study between those tissues. Here we are hypothesizing that LRRK2 interacts differently in different tissues (Lewis and Manzoni, 2012) and these specific interactors might shed light on new avenues for LRRK2 targeted therapy with minimal off-target effects.

## 2 Results

### 2.1 LRRK2 interactome in the brain

**Fig. 1:**
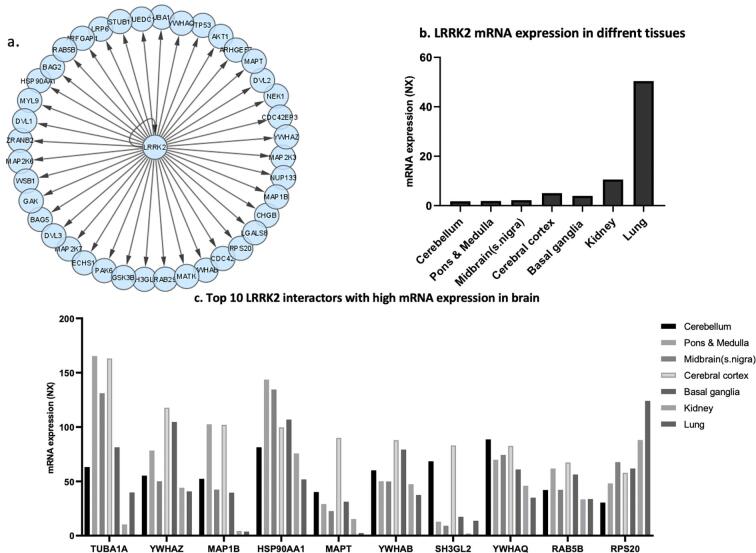
a) A visualization of LRRK2 interactome specific to brain tissue after applying filters- high confidence score and direct interactors. The graph shows LRRK2 as a center seed and its direct interactors as nodes (blue circles). b) Graph showing LRRK2 mRNA expression in lungs, kidney, and different brain regions; c) top 10 LRRK2 interactors with high mRNA expression levels in brain regions (basal ganglia, cerebral cortex, midbrain, pons/medulla, and cerebellum), compared to peripheral tissues – lung and kidney.

LRRK2 was queried for brain interactors with a high confidence score (>0.72) (Filter#1) in HIPPIE. We found 118 interactors including direct interactors, physical association, association, and colocalization (Filter#2). Upon application of interactions in all brain tissues (Filter#3), the number of nodes reduced to 115. Further filtering for direct interactions in brain tissue the number of interactors was reduced to 42 (Fig. 1a). To assess the relative prominence of each of the 42 LRRK2 interactors in the brain, and assuming that more highly expressed genes will have a greater functional role in a given tissue, we investigated the mRNA expression levels of the interactors in different brain tissues. Additionally, we investigated peripheral tissues, including kidney (10.6 NX) and lung (50.4 NX), where *LRRK2* expression is exceptionally higher than in brain tissues (average 2.94 NX) (Fig. 1b). As shown in Fig. 1c, expression levels of the top 10 most highly expressed interactors in the brain varied across brain regions – cerebellum, midbrain, pons/medulla, cortex, and basal ganglia along with kidney and lungs.

### 2.2 Comparison of LRRK2 PPI network across brain tissues

Based upon the variation in the mRNA expression levels of *LRRK2* gene and its interactors across brain regions, we created tissue-specific interactome for PD affected brain tissue- substantia nigra, frontal cortex, anterior cingulate, and basal ganglia along with the cerebellum as an unaffected brain tissue. We also created a LRRK2 interactome network in peripheral tissues specifically in the kidney and lungs due to the high endogenous expression of *LRRK2*. For creating these sub interactomes filter#1 and #2 were applied whereas filter #3 selected each tissue individually.

**Fig. 2:**
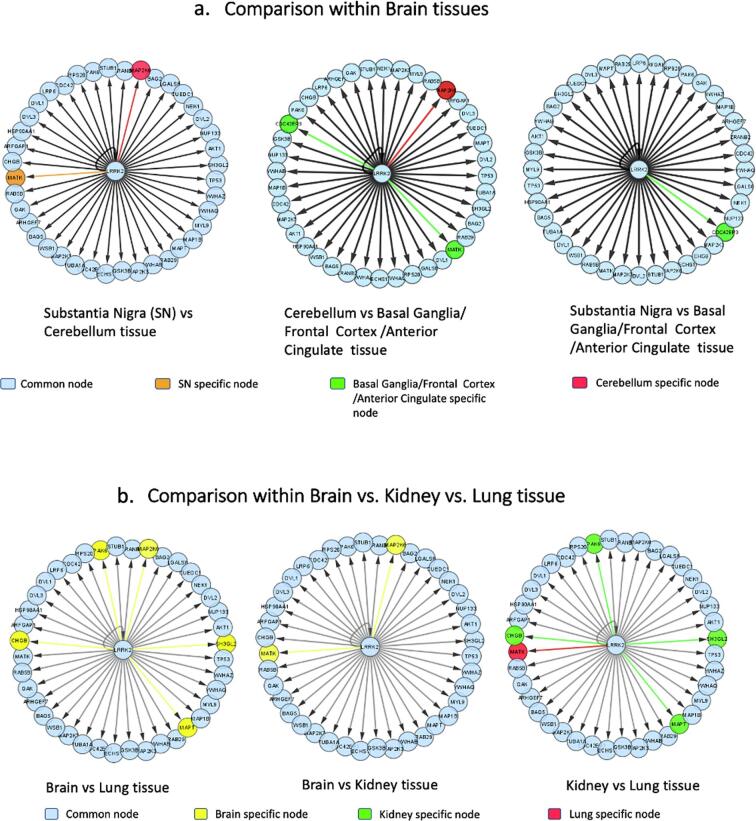
Comparative analysis of LRRK2 interactome in different tissues: a-c show the visualization of the comparative LRRK2 interactome within brain tissues - substantia nigra, basal ganglia, frontal cortex, anterior cingulate, and cerebellum. To be noted that LRRK2 interactome in basal ganglia, frontal cortex, and anterior cingulate was identical; d-f show the visualization of comparative LRRK2 interactome among brain, kidney, and lung tissues. The most rewired nodes have been color-coded (see the legends) for each section.

The LRRK2 interactome for substantia nigra and cerebellum had 39 (nodes) direct interactors whereas 40 nodes were seen in the frontal cortex, anterior cingulate, and basal ganglia network, which shared the same LRRK2 interactome. A comparison of these tissue-specific LRRK2 networks with each other using DyNet showed that there were 38 common nodes in all the 5 selected brain tissues (Fig. 2). Interestingly, as shown in Fig. 2 (a-c), it was found that LRRK2 specifically interacts with- (i) CDC42 effector protein 3 (CDC42EP3) in basal ganglia, frontal cortex, and anterior cingulate, (ii) Megakaryocyte-associated tyrosine-protein kinase (MATK) in substantia nigra, basal ganglia, frontal cortex, and anterior cingulate, and (iii) Mitogen-activated protein kinase 6 (MAP2K6) in the cerebellum only.

**Fig. 3:**
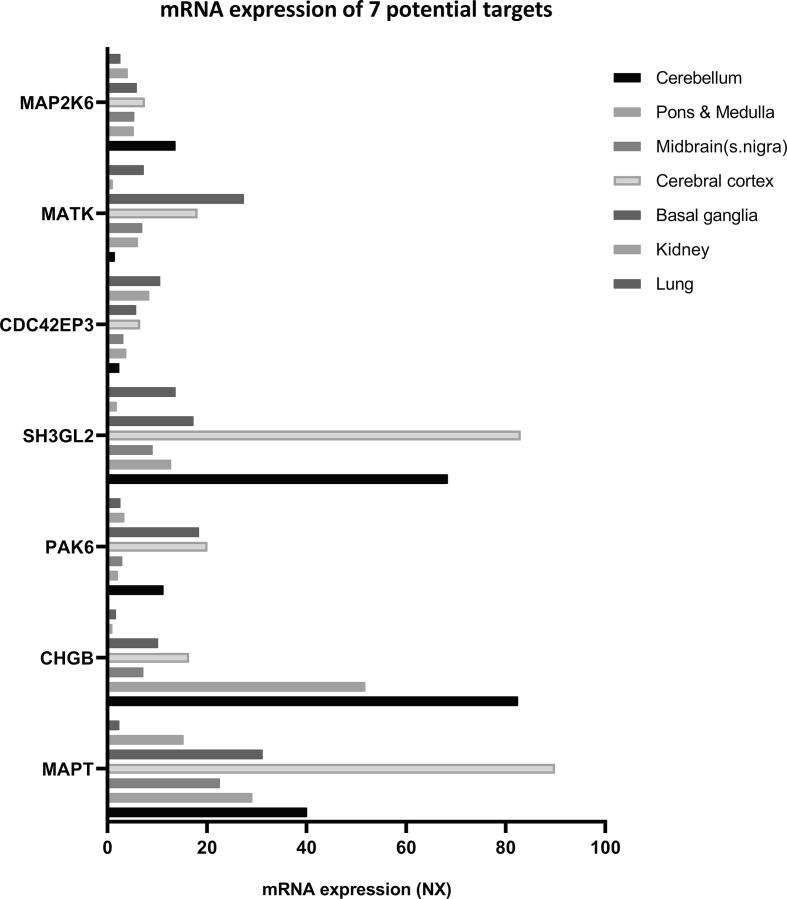
mRNA expression levels of LRRK2 interactors that interacts differently in the brain, kidney, and lung tissue.

Further, the mRNA expression levels of *CDC42EP3, MATK, MAP2K6* were checked within brain tissues. As shown in Fig. 3, *CDC42EP3* was expressed more in the basal ganglia and cerebral cortex, when compared to the medulla, substantia nigra and cerebellum. *MATK* was expressed least (1.5 NX) and *MAP2K6* highest (13.7 NX) in the cerebellum as compared to other brain tissues. Hence, these findings suggest that LRRK2 interacts differently in different tissues based on the mRNA expression level of interactors.

### 2.3 Comparison LRRK2 PPI networks in brain, kidney, and lung

Next, we created LRRK2 interactome for direct interactors in lung and kidney tissue individually. 39 and 36 nodes were found in kidney and lung tissue interactome, respectively. On comparing these two LRRK2 interactomes with brain tissue interactome composed of 42 interactors, we found 36 common LRRK2 interactors are present in the brain, lung, and kidney tissues (Fig. 2). Notably, it was found that MAPT, CHGB, PAK6, and SH3GL2 interacted with LRRK2 in the brain and kidney but not in lung tissue. Also, MATK interaction with LRRK2 was absent in kidney tissues whereas LRRK2 showed interaction with MAP2K6 only in brain tissues.

Further, we investigated the expression level of these 7 interactors within brain regions as well as kidney and lung tissue (Fig. 3). The expression levels of *MAP2K6*, *MAPT*, and *CHGB* were exceptionally low in kidney and lung tissues when compared to brain tissues especially the cerebral cortex, cerebellum, and pons/medulla. Additionally, at the protein level, CHGB was not detected in kidney or lungs (Supplementary Table S1). *MAPT* mRNA levels were more highly expressed in the cerebral cortex amongst the brain regions examined followed by kidney and lung tissues. *PAK6* levels were comparable in the cerebellum, cerebral cortex, and basal ganglia (16.6 NX) but higher than in pons/medulla, midbrain, kidney, and lungs in which *PAK6* levels were almost equal (between ~2 and 3 NX). *CDC42EP3 *expression levels were relatively low across the board with higher expression in the lungs and kidneys compared to all brain regions. Additionally, *MATK *expression levels were lowest in the kidney and cerebellum (~1.1 and 1.5 NX). However, both *MATK* and *SH3GL2* showed exceptionally high expression in the cerebral cortex with lower expression in the lungs and kidneys. These were also found to have low expression at the protein level (Supplementary Table S1). Altogether, this study has shown seven potential targets for tissue-based LRRK2 interactions and targeted therapeutics. Table 1 shows the summary of these 7 interactors including methods used for detection.

**Table 1:** Synopsis of the 7 LRRK2 specific interactors identified in the study.

Gene	Protein	Uniprot ID	Detection method	LRRK2 Domain	References
MAP2K6	Dual specificity mitogen-activated protein kinase kinase 6	P52564	affinity chromatography technology, anti-tag coimmunoprecipitation, enzymatic study, fluorescence microscopy	Kinase and COR	(Hsu et al., 2010, Gloeckner et al., 2009)
MATK	Megakaryocyte-associated tyrosine-protein kinase	P42679	protein array, pull down	Full length	(Tomkins et al., 2018, Beilina et al., 2014)
CDC42EP3	Cdc42 effector protein 3	Q9UKI2	anti-tag coimmunoprecipitation, affinity chromatography technology	ROC-COR Kinase Domains	(Haebig et al., 2010, Chan et al., 2011)
MAPT	Human-Tau	P10636	protein kinase assay, anti-tag coimmunoprecipitation, affinity chromatography technology, pull down, enzymatic study	Full length	(Kawakami et al., 2012, Bailey et al., 2013)
CHGB	Secretogranin-1	P05060	protein array, pull down	Full length	(Reyniers et al., 2014, Beilina et al., 2014)
PAK6	Serine/threonine-protein kinase PAK 6	Q9NQU5	protein array, pull down	Full length	(Reyniers et al., 2014, Beilina et al., 2014)
SH3GL2	Endophilin-A1	Q99962	protein kinase assay, enzymatic study	Kinase	(Arranz et al., 2015, Matta et al., 2012)

To better understand the relationship between the genes encoding the LRRK2 interacting proteins shown in table 1, we additionally investigated how they cluster in gene co-expression networks across multiple brain and peripheral tissues. We found that *LRRK2, MAP2K6*, *MATK*, *CDC42EP3*, *CHGB*, *PAK6*, and *SH3GL2* cluster in fewer co-expression modules in brain regions affected in PD (e.g. substantia nigra, caudate and putamen; up to 4 genes in the same module) when compared to the lung tissue (all genes in different modules, Supplementary Table S2), suggesting they are involved in related pathways/functions (e.g. chemical synaptic transmission, Supplementary Table S2) in those brain region but not in the lung. It is of note that in the substantia nigra, *CHGB*, *MATK*, and *SH3GL2* cluster in a co-expression module which is enriched for neurons, including dopaminergic neurons (Supplementary Table S2), supporting an important role for these genes in this particular cell type.

### 2.4 Functional enrichment analysis

**Fig. 4:**
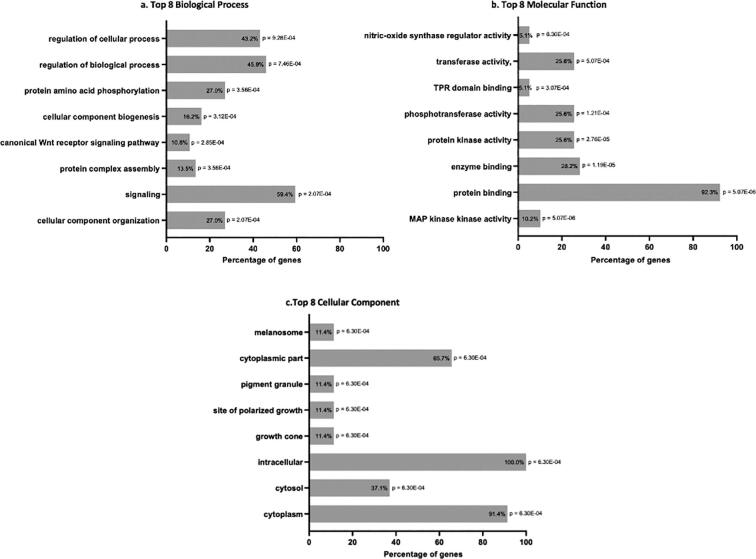
GO enrichment analysis using BiNGO: The graphs represent GO enrichment analysis results (top 8 GO terms) for the a) biological processes of the LRRK2 interactome, b) molecular functions, and c) cell components.

To check whether these tissue-specific interactions of LRRK2 associate with any tissue-specific functions we also performed GO enrichment analysis for the biological processes and molecular functions (Fig. 4). LRRK2 was expressed abundantly in all brain regions (Fig. 1b), hence it may have a conserved role in an essential cellular process across the brain. All the 42 LRRK2 interactors found in the brain tissue were used as a gene set for enrichment analysis of Gene Ontology (GO) terms: biological process, molecular function, and cell components using BiNGO. For all the three GO enrichment terms, we have shown here the top 8 GO terms (Fig. 4), the detailed results of this analysis is present in Supplementary Table S3 Moreover, we coupled together similar types of GO terms, for example, signaling transduction and transmission were paired into signaling, to cover more processes.

Upon carrying out biological process enrichment for LRRK2 and its interactors, 424 GO terms were significantly enriched. The predominantly enriched “GO biological process” term included signaling, cellular component organization and biogenesis, protein complex assembly, and amino acid phosphorylation (Fig. 4a). Most of the interactors were involved in signaling (59.4%), regulating biological processes (45.9%), and cellular processes (43.2%). On the other hand, there were a total of 77 GO molecular function terms significantly enriched for LRRK2 and its interactors. The top molecular functions included kinase activity, protein and enzyme binding, phosphotransferase activity, and nitric oxide regulatory activity (Fig. 4b). Most interactors were involved in protein binding (92.3%), enzyme binding (28.2%), kinase activity (25.6%), and transferase activity (25.6%).

GO cell component enrichment showed that all the LRRK2 interactors in interactome are intracellular proteins, mostly present in the cytoplasm and cytosol. Amongst this, 11.4% of interactors are specifically found in the growth cone, site of polarized growth, and pigment granules (Fig. 4c).

We also performed GO enrichment for sub interactomes in brain tissue and kidney and lung. It was observed that number of enriched GO terms varied but the top hits remained the same, as most interactors are shared across tissues. Full list of GO analysis is provided in Supplementary Table S3.

## 3 Discussion

Developing disease modifying therapies for PD remains a critical unmet medical need. In this regard, LRRK2 is considered a priority therapeutic target for both forms of PD, familial and idiopathic since genetic, molecular, and pre-clinical studies support the involvement of LRRK2 in the pathophysiology of PD, with a missense mutation in *LRRK2* being the most common cause of familial PD and common *LRRK2* variants acting as risk factors for idiopathic PD (Klein and Westenberger, 2012). The precise function of LRRK2 is still unknown, but due to the presence of its dual enzymatic activities and other protein–protein interaction domains it is involved in a myriad of cellular functions including autophagy, lysosomal functions, signaling, and also immune functions (Gomez-Suaga et al., 2014, Wallings et al., 2015). Moreover, LRRK2 based therapies are currently at advanced clinical trial phase (NCT04056689 and NCT03976349).

A number of studies have investigated the impact of reducing LRRK2 activity in animal models. These treatments including genetic knockout of LRRK2 and the use of LRRK2 kinase inhibitors like- GNE-7915 and GNE-0877 contributed to pathological phenotype in the liver, lungs, and kidney of rodent and non-human primate models (Andersen et al., 2018, Baptista et al., 2013, Fuji et al., 2015, Herzig et al., 2011, Ness et al., 2013) as side effects. Although further recent studies showed LRRK2 inhibitors like GNE-7915 or MLi-2 have a reversible effect on lung phenotype after withdrawal of drugs in non-human primates (Baptista et al., 2020) and a partial reduction of LRRK2 protein levels caused by loss of function variants is well tolerated in humans and does not cause severe clinical phenotypes (Blauwendraat et al., 2018, Whiffin et al., 2020). Therefore, it is clear that LRRK2 therapy is a viable option for PD therapy provided the side effects on peripheral organs can be managed. Our study using an *in silico* approach to investigate differential LRRK2 protein interactions in different brain regions and in peripheral tissues with high LRRK2 expression has identified 7 potential LRRK2 interactors which should further help to define the role of LRRK2 and its potential targets to modulate LRRK2 activity, without affecting peripheral organ activity. The result of such computational analysis will further foster possible wet-lab investigations and will provide complementary data for testing the efficacy of such treatments.

For the purposes of this study, a brain-specific LRRK2 network was generated, based on previous PPI literature data present in HIPPIE up to 30th June 2020. Further sub-interactomes were created separately examining the following brain regions substantia nigra, basal ganglia, cerebellum, frontal cortex, and anterior cingulate. We also created LRRK2 protein interactomes for lung and kidney as LRRK2 expression levels are higher in these tissues (Fig. 1b). After filtering the data, altogether there were 42 direct interactors of LRRK2 in the brain (Fig. 1a) out of which the substantia nigra and cerebellum had 39 interactors while the rest of the tissue had 40 interactors. In contrast, there were 39 and 36 direct interactors of LRRK2 in kidney and lung tissues, respectively. Further, results from a comparison of these tissue-specific LRRK2 interactomes (Fig. 2) along with mRNA expression of all 42 interactors in different brain tissues, kidney, and lungs we show that LRRK2 interacts differently in different tissues (Fig. 1c), suggesting organ-specific interactions. Interestingly for PD, co-expression analysis of mRNAs revealed that 3 of our LRRK2 interactors *CHGB*, *MATK* and *SH3GL2* cluster together in dopaminergic neurons (Supplementary Table S2).

GO enrichment analysis using BiNGO showed involvement of LRRK2 interactors in following enriched functions (Fig. 4) - cellular component organization (27%), signaling (59.4%), protein kinase activity (25.6%), protein binding (92.3%) and, enzyme binding (28.2%). LRRK2 being an active kinase it further regulates other kinases in its downstream cascade, regulation of other protein kinase activities was expected. Although at present it is unclear whether the 9 further LRRK2 interactors (AKT1, GAK, GSK3B, NEK1, MAP2K3, MAP2K6, MAP2K7, MATK, PAK6) with enriched in kinase activity (Supplementary Table S3 – molecular function) regulate LRRK2′s kinase activity or *vice versa*. Further, 10.8% of LRRK2 interactome were involved in the canonical Wnt receptor signaling pathway, further supporting a role for LRRK2 in bridging membrane and cytosolic components of Wnt signaling (Berwick and Harvey, 2012). Interestingly, canonical Wnt receptor signaling pathway has been linked with PD (Daniel and Harvey, 2012). A study showed that pathogenic LRRK2 causes abnormal Wnt signaling pathways, further inhibiting LRRK2 kinase activity using LRRK2-IN-1 showed similar impairment (Berwick and Harvey, 2012). Hence, we can also target the Wnt signaling pathway as a putative therapeutic strategy for PD. Additionally, 10 interactors were also involved in phosphotransferase activity (Supplementary Table S3).

MAP2K6 is known to activate PAK6, together they are actively involved in protein kinase activity, regulation of transcription, and apoptosis. Moreover, PAK6 acts as a regulator for LRRK2 kinase activity by regulating 14-3-3γ (LRRK2 direct interactor) phosphorylation (Civiero et al., 2017). A study showed activated PAK6 rescues G2019S LRRK2 mutation related phenotype like neurite shortening (Civiero et al., 2017). Additionally, MAP2K6 has been implicated in the regulation of LRRK2 protein expression level (Hsu et al., 2010). Hence MAP2K6 and PAK6 might have the potential for regulating LRRK2 kinase activity along with the cell death pathway in PD (Iaccarino et al., 2007). Despite its function, MAP2K6 might not be a good candidate for potential therapy as it interacts with LRRK2 only in the cerebellum, a region that is not clinically or pathologically affected in PD. Further, one needs to be aware of the effects on the MAPK pathway by targeting MAP2K6. Whereas we think that PAK6 is a slightly more valid target for PD therapies as it interacts in all 5 brain regions although it is a direct interactor of LRRK2 in the kidney.

MATK participates in signal transduction in hematopoietic cells and has an inhibitory role in the control of T-cell proliferation. Interestingly recent studies have hypothesized the neuroprotective role of T cell in PD brains (Baird et al., 2019, Garretti et al., 2019), hence MATK can be used to target autoimmune pathway for PD investigations. Moreover, MATK is not an interactor of LRRK2 in kidney tissue as well as its mRNA expression exceptionally low in kidney (1.1 NX), nevertheless, it is an interactor in the lung and its mRNA expression is comparable to brain tissues, thus it might not be the best potential target since it can show lung phenotype. CDC42EP3 is also not a good candidate since it does not display interaction with LRRK2 in substantia nigra. Moreover, it also has low expression levels in the brain. However, PAK6 and CDC42EP3 are known to involve in cytoskeleton regulation (Farrugia and Calvo, 2017, Molli et al., 2009) hence they might have a role in the regulation of endocytic pathways in PD.

In contrast, MAPT, SH3GL2, and CHGB are strong candidates for LRRK2 targeted research. MAPT and CHGB both are highly expressed in brain regions, and showed interactions with LRRK2 in all 5 selected brain regions. Although these 3 targets do not interact with LRRK2 in lung tissue they do interact with LRRK2 in the kidney, albeit with relatively low mRNA and protein expression in the kidney and lungs. *MAPT* encodes for human tau protein, a key component of pathology in Alzheimer’s disease (AD), and it is also linked with PD as a genetic risk factor (Jensen et al., 1999, Vincent et al., 2018). Moreover, a recent study showed LRRK2 mediated endocytosis as one of the major pathways for tau spreading *in vivo*. Inhibition of LRRK2 kinase activity reduced neuronal uptake of monomeric and aggregated tau (Evans et al., 2020). Common variation at the LRRK2 locus has also been linked with disease progression in a primary tauopathy, progressive supranuclear palsy (PSP) (Jabbari et al., 2020). Additionally, studies have associated tau pathology with LRRK2 mutations (Henderson et al., 2019, Zimprich et al., 2004) whilst another study demonstrated that LRRK2 phosphorylates tau and promotes tauopathy (Bailey et al., 2013). MAPT mediates microtubule assembly, apoptosis, astrocyte activation, and chaperone binding. All these functions have been linked with impairment in autophagy and neuroinflammation in PD (Tait and Green, 2010, Waak et al., 2009) thus, making MAPT as a good potential target for LRRK2 pathway investigations and further connecting LRRK2 with PSP and AD.

CHGB is potentially the best target with regard to brain specificity among the 7 interactors since it has very low expression in kidney and lung tissues (1 and 1.7 NX respectively) and also at protein levels compared to other potential targets. It is also worth noting that this pattern of expression appeared to be consistent at the protein level. So, targeting CHGB might have the least side effects. Moreover, CHGB is a neuroendocrine secretory granule protein that mediates cellular protein metabolism and their post-translational modification (PTM). Hence, CHGB might be able to modulate LRRK2 kinase activity as well as PTM of pathological alpha-synuclein (Shahpasandzadeh et al., 2014, Zhang et al., 2019) in PD to prevent disease progression.

Whereas SH3GL2, coding for endophilin 1A and is also a candidate risk factor for PD (Bandres-Ciga et al., 2019), interacts with LRRK2 and is highly expressed in brain tissue and potentially could be an important molecule for future research on LRRK2 based investigations. Furthermore, co-expression network analysis suggests that *SH3GL2* is important for dopaminergic neurons. Our analysis shows it is a direct LRRK2 interactor in the kidney but not in the lungs, although its expression level is less in the kidney. So, targeting it may or may not have major side effects on these peripheral tissues. Moreover, it is involved in synaptic vesicle endocytosis and protein kinase activity, both these functions are highly linked to PD (Piccoli et al., 2011, Shin et al., 2008). Additionally, LRRK2 has been reported to mediate endophilin 1A phosphorylation in a kinase-dependent manner, which in turn leads to synaptic vesicle endocytosis (Arranz et al., 2015, Matta et al., 2012). Hence, SH3GL2 might modulate the endocytosis pathway to prevent PD progression.

Overall, these seven LRRK2 interactors are involved in endocytosis, autophagy, and vesicle transport pathways, all of which are impaired in PD. Hence, these potential targets might play individual roles in PD development. One idea of using these potential targets is that they could act as a modifier/modulator of LRRK2 activity. Since the LRRK2 kinase activity is elevated in PD linked to LRRK2 mutations and common genetic variants are involved in the idiopathic form of PD (Di Maio et al., 2018), it would be good to find a way to modulate LRRK2 kinase activity to normal optimal physiological levels rather than inhibiting it completely, since LRRK2 might be involved in other important functions within cells. For example, LRRK2 kinase activity is important for synaptic vesicle endocytosis and subsequent neurotransmission at the synapse, with inhibition of LRRK2 causing impairment of these (Arranz et al., 2015). The potential targets identified by this study provide a starting point to find modulators of LRRK2 kinase activity. For example, MAP2K6 and PAK6 can modulate LRRK2 kinase activity (Civiero et al., 2017, Hsu et al., 2010). Further experimental studies must be conducted with these potential targets to see how they modulate LRRK2 function and to which LRRK2 domain they interact with, and importantly whether they also interact with mutated LRRK2. Thus, the result gain from this would open new avenues for LRRK2 targeted investigations for PD. Additionally, this *in silico* approach can help to predict the on and off-target effect of modulating LRRK2 activity in different tissues. Another idea towards developing a LRRK2 targeted therapy could be that, since LRRK2 is a complex protein, it might be difficult to modify its pathogenic activity directly instead we can target the impaired PD pathways associated with LRRK2 by targeting these interactors. For example, the apoptosis pathway can be modulated by PAK6 (Zhang et al., 2010), MAP2K6 (Bragado et al., 2007, Rasmussen et al., 2016), and MAPT (Wang et al., 2010, Zaman et al., 2018). Moreover, based on GO enrichment highlighting canonical Wnt signaling could also be a potential therapeutic pathway. In the future, interactors of these potential targets could be investigated to find any novel pathway or protein linking to PD related investigations.

HIPPIE provides a powerful tool for investigating protein interactomes as it provides highly curated data from published literature, along with different filters especially MI scoring. However, HIPPIE still has some limitations. Although MI/confidence scoring confirms an interaction based on the number and type of techniques used, the number of organisms used to test interaction, and the number of published works, it lacks demonstration of the strength of the interactions. Due to this drawback, some proteins might be a strong interactor of LRRK2, but due to lack of research done on them they score a low MI score, hence might not be included in the interactome (Gloeckner and Porras, 2020). This might be the reason that the expression level of SH3GL2 is least expressed in the kidney (1.9 NX) and it interacts with LRRK2, whereas in the lung its expression is 13.7 NX, yet it is not annotated as an interactor. Similarly, high confidence score interactors might have weak interaction. Also, weighted scoring based on techniques used for example techniques like protein assays or arrays are scored higher than yeast two-hybrid, thus the scoring lacks resolution at the level of the experimental detail for specific interactions. Hence interactions of these potential targets need to be further explored and validated both *in vivo* and *in vitro* paradigms. This taken together shows the clear limitations surrounding using data on interactions reported from various sources using different methodologies.

PPI databases are dependent on data available and as such might be skewed towards proteins and/ or tissues that are of higher interest to the research community. Additionally, manual curation of the HIPPIE database is time consuming resulting in an invariable lag in updating the database. Hence, these *in silico* methods generate hypothesis that needs to be tested experimentally.

Additionally, due to the lack of reliable tissue specific protein data, we have used mRNA expression as a proxy for protein expression. We are aware that there may be post-transcriptional factors modulating protein level expression. We did indeed use protein expression levels based on scoring provided by the Human Protein Atlas to validate our findings whenever possible. These expression scores are also often provided by either mass spectrometry data or immunohistochemistry data leading to some variability in expression scores depending on the method. It is also worth noting that protein expression data is incomplete for most brain regions making tissue specific analysis based on this data difficult.

In the future, it would be useful to examine additional tissues as LRRK2 is involved in IBD (Jostins et al., 2012), leprosy (Zhang et al., 2009), and cancer (Ruiz-Martínez et al., 2014, Saunders-Pullman et al., 2010). Moreover, as LRRK2 expression is elevated in immune cells in PD (Cook et al., 2017, Herbst et al., 2020) it might be valuable to perform computational analysis in a cell-specific manner. Unfortunately, to date there is not much data to investigate in other tissues and cell types.

In conclusion, our comparative study of LRRK2 interactomes from brain, kidney, and lung tissue is the first analysis of its kind and depicts that LRRK2 interacts differently in different tissues. These tissue-specific interactions could shed light on the tissue-specific function of LRRK2, which might provide insights as to why only a few brain regions are affected in PD. Interestingly, few proteins in the LRRK2 interactome have also been suggested to be genetic risk factors for PD like GAK and SH3GL2. Our computational analysis has given us 7 potential targets for LRRK2 based therapy that are less likely to have side-effects in peripheral organs. The enrichment analysis of LRRK2 interactomes showed different processes and functions in which LRRK2 is involved, this further provides clues on the mechanisms by which LRRK2 may cause PD. Importantly, the above discussed *in silico* approach provides a good starting point for hypothesis-driven wet laboratory-based investigations and an effective method to study potential on- and off-target effects for drug targets. Our work should stimulate further studies aiming to validate tissue specific LRRK2 interactions, develop LRRK2 based therapy and identifying the biological function of LRRK2. This sort of analysis could also be extended to other Parkinson’s disease genes as well as to other neurodegenerative diseases.

## 4 Experimental procedure

### 4.1 Construction of LRRK2 interactome: Human Integrated Protein-Protein Interaction rEference (HIPPIE)

LRRK2 (human, Q5S007) PPI network was downloaded from HIPPIE v2.0 (Alanis-Lobato et al., 2017) (http://cbdm-01.zdv.uni-mainz.de/~mschaefer/hippie/network.php, accessed in June 2020) under the network query section in PSI-MI 2.5 TAB format and was imported to Cytoscape v3.8.0 for visualization. HIPPIE integrates PPI data from several databases, including IntAct (Orchard et al., 2014), BioGRID (Chatr-Aryamontri et al., 2017), Human Protein Reference Database (HPRD) (Keshava Prasad et al., 2009), Database of Interacting Proteins (DIP) (Salwinski, 2004), Biomolecular Interaction Network Database (BIND) (Isserlin et al., 2011), Mammalian Protein-Protein Interaction Database (MIPS) (Pagel et al., 2005), and The Molecular INTeraction Database (MINT) (Chatr-Aryamontri et al., 2007). In HIPPIE, all PPI data were merged, underwent quality control and filtering based on the International Molecular Exchange (IMEx) consortium (Orchard et al., 2012) curation rules (Curation < IMEx (imexconsortium.org) to remove any redundancy in database, for example each paper should be present only once in the IMEx dataset. Only human and experimentally proven interactions were retained. For tissue-specific networks, gene expression data from Genotype-Tissue Expression (GTEx) (Ardlie et al., 2015) were merged with the PPI network, thus nodes representing non-expressed genes in the selected tissue were excluded from the network (Alanis-Lobato et al., 2017). Finally, HIPPIE performs scoring (0 to 1) which reflects the reliability of PPI, based on experimental techniques used, the number of studies finding the PPI, and reproducibility in model organisms (Schaefer et al., 2012).

For creating tissue-specific LRRK2 interactome we applied the following filters in HIPPIE. Filter#1 High confidence score i.e. confidence score of >0.72 was set to get the bonafide LRRK2 interactors. Filter#2 Interaction type: Direct interactors (based on experiments performed between only two pure proteins for example as in yeast two hybrid assays). Filter#3 was applied one at a time to choose the tissue – brain (all), substantia nigra, cerebellum, basal ganglia, anterior cingulate and frontal cortex, kidney, and lung. A total of seven interactomes were created individually.

### 4.2 Comparison between tissue-specific LRRK2 interactome

A comparison between the tissue-specific LRRK2 interactomes was performed using the Cytoscape plug-in app DyNet analyzer (Goenawan et al., 2016). Biefly, DyNet identifies and analyzes the most rewired nodes/edges, based on their presence/absence or the value of a selected numeric attribute (e.g. node abundance, edge weight), between two or more networks and displays them with differential color coding on the central reference network (merged network) and as an added attribute in Cytoscape’s table view.

For comparing the presence/absence of nodes Dynet superimpose all the nodes from one network over another, the absent/present node is highlighted as a result.

### 4.3 LRRK2 interactome mRNA expression across tissues

mRNA expression of every LRRK2 interactor within the brain, kidney, and lung tissues were taken independently from the Human Protein Atlas (HPA, https://www.proteinatlas.org/). For this study, we captured the consensus normalized expression (NX) data, which includes RNA sequence data from three different sources: internally generated Human Protein Atlas (Uhlen et al., 2015), GTEx (Ardlie et al., 2015), and CAGE data from FANTOM5 project (Consortium et al., 2014).

Additionally, for the *LRRK2* gene and the seven potential targets for tissue-based LRRK2 interactions (*MAP2K6*, *MATK*, *CDC42EP3*, *MAPT*, *CHGB*, *PAK6*, and *SH3GL2*) we investigated how these genes cluster at the mRNA level by investigating gene co-expression networks with the CoExp WebPage tool (https://rytenlab.com/coexp, (Botía et al., 2017)). We have used GTEx V6 networks, which include co-expression networks in control brain and peripheral tissues of interest, namely substantia nigra, basal ganglia (caudate and putamen), frontal cortex, cerebellum and lung. All genes with the exception of *MAPT* were present in these networks. The CoExp WebPage tool also provides annotations of enriched GO terms and cell types for each co-expression module.

Given that mRNA expression across tissues can differ from protein level expression, protein expression data was also investigated whenever possible using the Human Protein Atlas expression scores. Protein expression scores are based on a best estimate of the “true” protein expression from a knowledge-based annotation. This is achieved by stringent evaluation of immunohistochemical staining pattern from 3 different antibodies and, a collective score is set displaying the estimated true protein expression.

### 4.4 Functional enrichment analysis

Gene ontology (GO) enrichment analysis was performed for all 8 interactomes using Cytoscape plug-in BiNGO (https://www.psb.ugent.be/cbd/papers/BiNGO/Home.html) (Maere et al., 2005). The whole *Homo sapiens* genome set was used as a reference set. GO enrichment dataset for biological process, molecular functions, and cell components was used individually. The analysis was performed using hypergeometric statistics and Benjamin and Hochberg False discovery correction for multiple testing. Adjusted p value < 0.05 was set as the significance threshold and overrepresented categories were selected for visualization. Analysis and visualization of the network were done using Cytoscape v3.8.0. All graphs were made using GraphPad by Prism 8. Details about proteins were taken from UniProt (https://www.uniprot.org/).

## CRediT authorship contribution statement

**Amrita Verma:** Conceptualization, Data curation, Formal analysis, Writing - original draft. **Kirsten Ebanks:** Conceptualization, Methodology, Supervision, Writing - review & editing. **Chi-Yee Fok:** Writing - review & editing. **Patrick A. Lewis:** Conceptualization, Supervision, Writing - review & editing. **Conceicao Bettencourt:** Investigation, Validation, Writing - review & editing. **Rina Bandopadhyay:** Conceptualization, Investigation, Project administration, Supervision, Writing - review & editing.

## Appendix A Supplementary data

Supplementary Table 1 Table showing protein expression scores from the Human Protein Atlas of the 7 interactors identified in the study.

Supplementary Table 2 Table of co-expression networks of the 7 interactors identified in the study.

Supplementary Table 3 Table for each GO component (biological, molecular and cellular) of the enriched terms: GO term and ID, p-value adjusted for multiple testing, number of LRRK2 interactors associated with the GO term (Genes), and frequency of genes contributing to each GO term in the interactome.
