# Supplementary figures and images for: In silico comparative analysis of LRRK2 interactomes from brain, kidney and lung
Source: Brain Res. 2021 Aug 15;1765:None. doi: 10.1016/j.brainres.2021.147503 (PMC8212912; doi:10.1016/j.brainres.2021.147503)

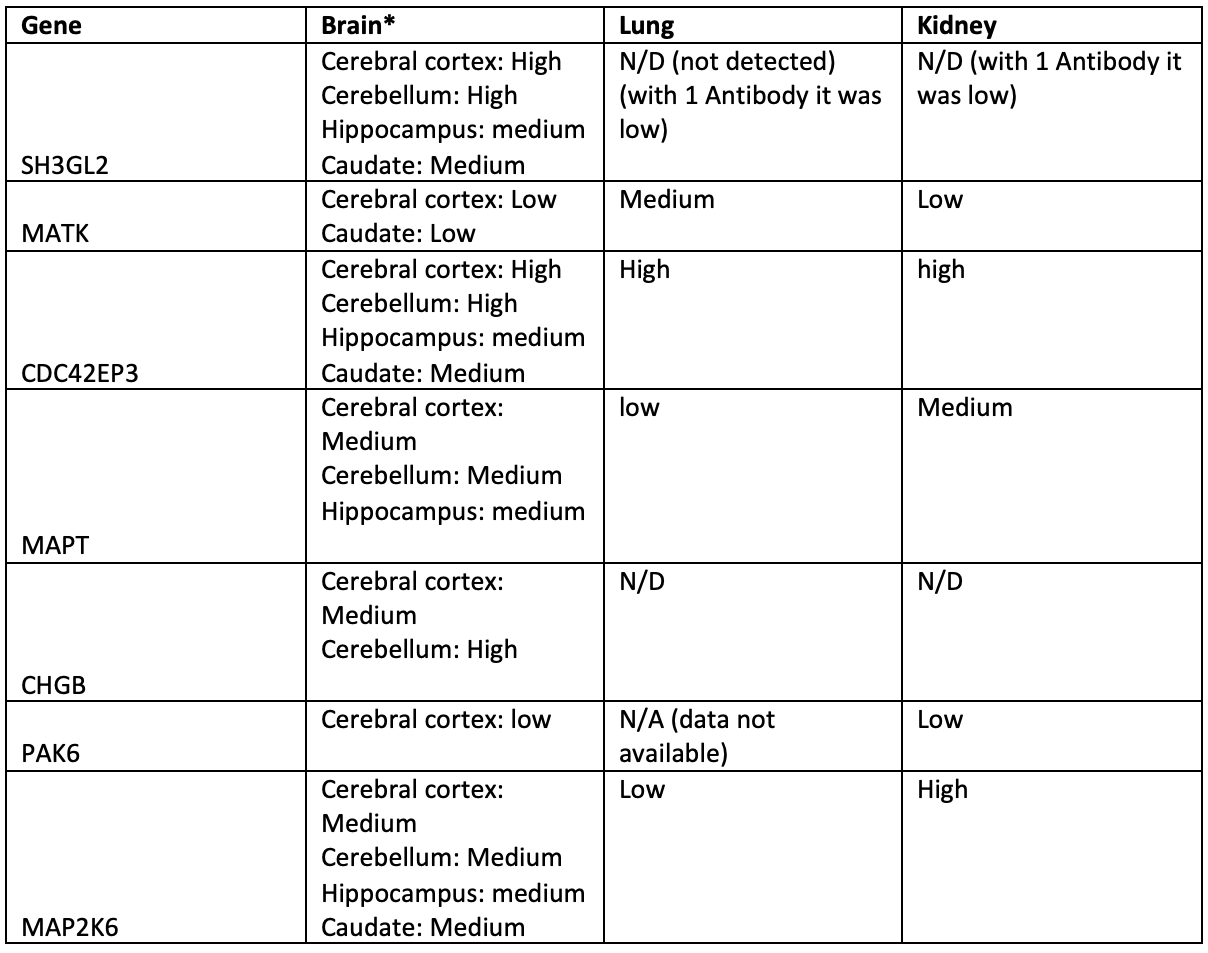

Supplement: Supplementary Table 1 — Table showing protein expression scores from the Human Protein Atlas of the 7 interactors identified in the study. [file mmc1.docx]
